# Laminin-5 is a biomarker of invasiveness in cervical adenocarcinoma

**DOI:** 10.1186/1746-1596-7-105

**Published:** 2012-08-17

**Authors:** Johji Imura, Yoshiaki Uchida, Kazuhiro Nomoto, Kazuhito Ichikawa, Shigeki Tomita, Tatsuo Iijima, Takahiro Fujimori

**Affiliations:** 1 Department of Diagnostic Pathology, Graduate School of Medicine and Pharmaceutical Sciences, University of Toyama, 2630 Sugitani, Toyama City, Toyama, 930-0194, Japan; 2 Department of Pathology, Ibaraki Prefectural Central Hospital, 6528 Koibuchi, Kasama, Ibaraki, 309-1793, Japan; 3 Department of Surgical and Molecular Pathology, Dokkyo Medical University School of Medicine, 880 Kitakobayashi, Mibu, Shimotsuga, Tochigi, 321-0293, Japan

**Keywords:** Cervical adenocarcinoma, Invasiveness, Laminin-5, Immunohistochemical analysis

## Abstract

**Background:**

Glandular lesions are often problematic for diagnostic cervical pathology. The survival of patients with adenocarcinoma is significantly poorer than that of patient with squamous cell carcinoma. One reason for this increased risk is the aggressive invasiveness of adenocarcinoma. Therefore additional biomarkers, to supplement morphological diagnosis of adenocarcinoma, are necessary. We have assessed the diagnostic utility of Laminin-5 (Laminin γ2 chain): Lam-5 in the diagnosis of the invasiveness of cervical adenocarcinoma and related glandular lesions.

**Methods:**

Lam-5 immunohistochemistry was performed on archival specimens from 8 patients with uterine leiomyoma as a negative control group, 6 patients with endocervical gland hyperplasia, 6 patients with adenocarcinoma in situ, 6 patients with microinvasive adenocarcinoma and 24 patients with invasive adenocarcinoma.

**Results:**

The expression of Lam-5 was not detected in normal mucosa, but was seen along the basement membrane in endocervical gland hyperplasia and adenocarcinoma in situ and was observed in the cytoplasm of tumor cells in microinvasive and invasive adenocarcinoma.

**Conclusion:**

We conclude that Lam-5 is a useful biomarker in the evaluation of invasiveness in cervical adenocarcinoma.

**Virtual slides:**

The virtual slides for this article can be found here: http://www.diagnosticpathology.diagnomx.eu/vs/7316562925827381

## Background

Although adenocarcinoma (AC) is rare, relative to squamous cell carcinoma (SCC), among uterine cervical cancers, the number of cases has increased in recent years, particularly in young women [1]. Like SCC, invasive AC is associated with high-risk human papillomavirus infection and arises from non-invasive precursors, namely cervical glandular intraepithelial neoplasia/adenocarcinoma in situ [2]. The distinction between AC and SCC is not only of academic interest since these glandular lesions present many problems for diagnostic cervical pathology. Many different types of benign glandular lesions of the endocervix increase the potential for cervical neoplasia [3]. However, the distinguishing features of AC and SCC have practical therapeutic and prognostic implications, since the survival of patients with AC is significantly poorer than that for patients with SCC. The high invasiveness of tumor cells is the main reason for poor prognosis in AC. Many factors affect the invasiveness of tumors. A wide variety of molecular markers have been evaluated as diagnostic tools in the identification of high-risk precursors of SCC and AC and the association with cell adhesion molecules has attracted attention recently. These molecules affect the protein constitution of the basement membrane and play an important role in invasiveness.

Laminin-5 (Laminin γ2 chain): Lam-5 consists of extracellular proteins and is among the components of the basement membrane. The major functions of Lam-5 include binding of epithelial cells to the basement membrane through the formation of hemidesmosomes [4] and the migration of epithelial cells during wound repair [5,6]. In addition, it has been reported that there is a correlation between its expression and tumor progression in various kinds of malignant tumors [7-20]. According to some reports, Lam-5 is found in front-line invasive tumor cells at the epithelial-stromal interface and plays an important role in cancer cell invasion [9,11,12,17-23]. Accumulating data indicates that Lam-5 expression can serve as a marker for invasiveness in carcinoma cells from different tissues [18,19,23]. In uterine cervical cancers, recent data suggests that expression of Lam-5 could be a useful marker in the detection of invasive squamous cell carcinoma [20]. A more interesting finding, perhaps is that Lam-5 seems to be expressed from the earliest stages, particularly in microinvasive lesions.

Lam-5 may therefore become a very useful biomarker of early invasion in the AC of the uterine cervix. The aim of the present study was to assess whether Lam-5 can be used for evaluation of tumor cell invasiveness in cervical AC and other precancerous lesions. We also investigated whether Lam-5 can be used as a marker of invasiveness in neoplastic lesions.

## Methods

### Cases

Each of our 50 cases were registered at the Department of Surgical and Molecular Pathology, Dokkyo University School of Medicine and the Department of Pathology, Ibaraki Prefectural Central Hospital and were available for clinicopathological and immunohistochemical analysis. The patients were staged clinically according to the criteria of the International Federation of Gynecology and Obstetrics: FIGO. Of the 42 tumors, 6 were glandular hyperplasia, 6 were adenocarcinoma in situ (TMN Stage 0), 6 were microinvasive adenocarcinoma (Stage Ia) and 24 were invasive carcinoma (Ib were 16 cases, II were 5 cases and III were 3 cases).

### Immunohistochemistry

The control specimens were normal tissues from 8 cases of hysterectomy with diagnosis based on uterine leiomyoma. We selected routinely-processed, formalin-fixed, paraffin-embedded tissue blocks and prepared 5-micrometer serial sections from the cut surface of the blocks. Mouse monoclonal antibody (clone D4B45, Chemicon 1:100) was used for the present immunohistochemical study. Immunoperoxidase reactions were performed using a Venta View automated immunostainer according to the manufacturer’s instructions. All cases were reviewed by two investigators (JI and TF), and were provided with a consensus on the pathological diagnoses and the assessment of immunoreactivity.

## Results

**Figure 1:**
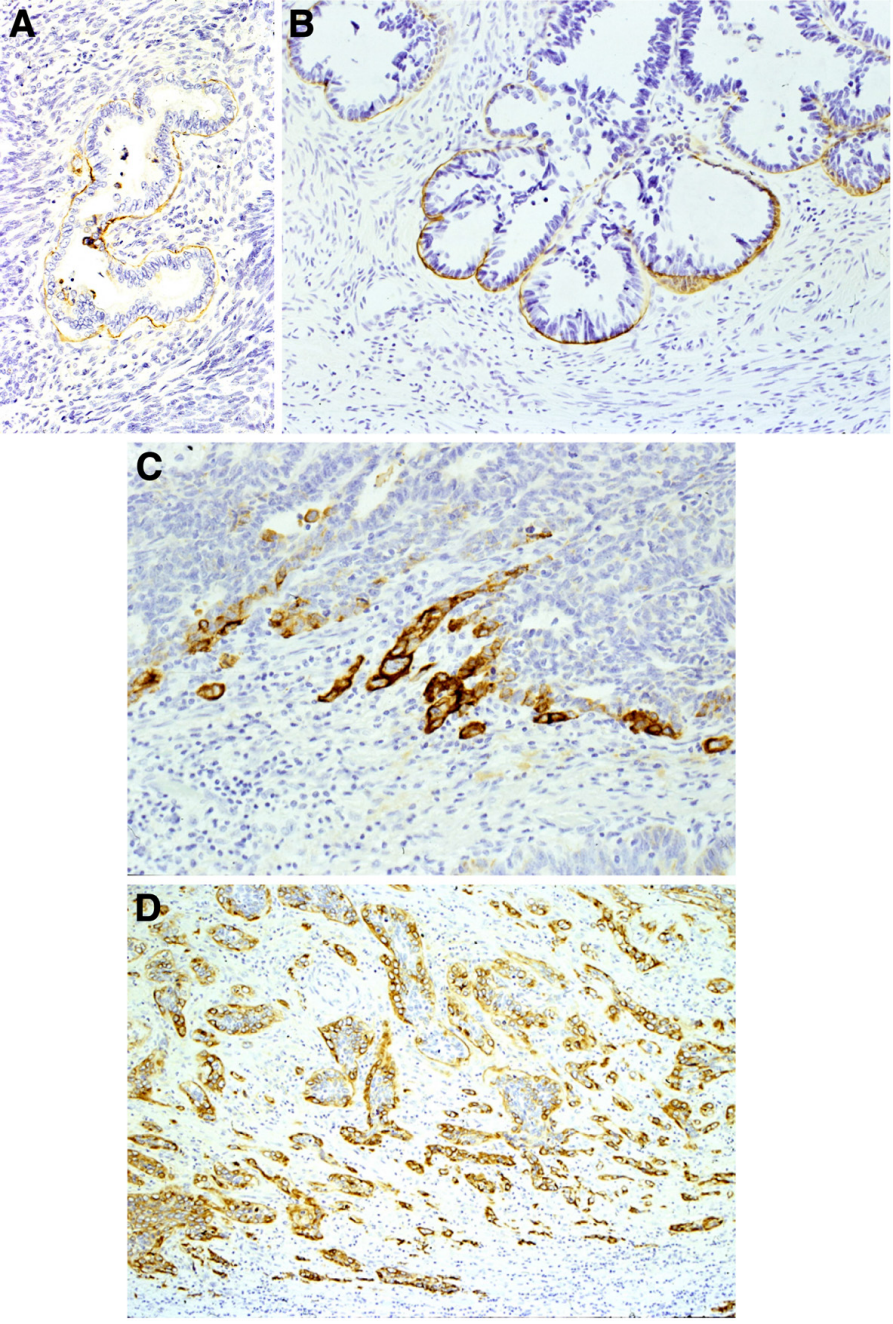
**Immunohistochemical findings for Laminin-5 in the normal mucosa and the lesions of uterine cervix.A**. Normal mucosa: No reaction in the endocervical glands. **B**. Endocervical gland hyperplasia and **C**. Adenocarcinoma in situ: Continuous and linear expression along basement membrane. **D**. Invasive adenocarcinoma: The diffuse immunoreactivity in most of tumor cells is scattered in the stroma.

No expression of Lam-5 was seen in normal endocervical glands and surrounding stroma (Figure 1A). Two of 6 cases (33%) of endocervical gland hyperplasia showed linear or focal basement membrane expression of Lam-5 (Figure 1B). Five of 6 (83%) cases of ’adenocarcinoma in situ’ showed continuous and linear Lam-5 expression along the basement membrane (Figure 1C). All of the 6 cases (100%) of microinvasive adenocarcinoma produced a positive reaction, with expression mainly present at the invading front. Four of these cases evidenced cytoplasmic expression with the remainder linearly expressed along the basement membrane. Cytoplasmic expression was specifically seen in budding or dissociated cells from tumor nests. Eighteen of 24 cases (75%) of invasive adenocarcinoma revealed cytoplasmic expression (Figure 1D). Cytoplasmic expression was diffuse in most tumor cells and scattered in the stroma. Linear expression along the basement membrane surrounding tumor cells was also observed in 11 of these cases. The immunohistochemical findings are summarized in Table 1.

**Table 1:** Association between for laminin5 γ 2 chain immunohistochemical expression and glandular lesions

**Histologic type**	**Case**	**Expression**	**Lamin-5**	
			**Pattern**	
			**Linear**	**Cytoplasm**
Normal	8	0	0	0
Hyperplasia	6	2	2	0
AIS	6	5	5	0
Microinvasive	6	6	2	4
Invasive	24	18	11	18

Normal: normal gland.

Hyperplasia: glandular hyperplasia.

AIS: adenocarcinoma in situ.

Microinvasive: microinvasive adenocarcinoma.

Invasive: invasive adenocarcinoma.

## Discussion

Laminins are a family of basement membrane proteins, which associate with cell differentiation proteins, adhesion and migration proteins, as well as being structural components themselves [24-26]. Lam-5 is a recently identified laminin isoform and is a disulfide-linked heterotrimer with precursor subunits of 200-, 155-, and 140-kDa. These precursors are rapidly processed by cleavage of the 200- and 155-kDa subchains into 165- and 105-kDd polypeptides, respectively [27]. Lam-5 is known to be expressed in squamous cell carcinoma, various adenocarcinomas and in organs such as the esophagus, cervix, breast, colon and pancreas [7-20]. An earlier study proposed that binding of the ligand to urinary-type plasminogen activator receptor: uPAR promotes cancer cell invasion by activation of plasminogen, leading to the degradation of extracellular matrix [28]. The coexpression of Lam-5 and uPAR suggested Lam-5 may be useful as a marker of invasion in some human cancers [18,23]. According to certain studies, Lam-5 is found at the invasive front, along the epithelial-stromal interface and plays an important role in cancer cell invasion [9,11,12,17-23]. Sordat et al. [23] and Pyke et al. [17,18] observed budding cancer cells linked with the accumulation of Lam-5 in the cytoplasm. In contrast, extracellular Lam-5 expression has been reported in gastric cancer and in basement membranes surrounding cancer cells [11]*.*

Lam-5 is expressed in hyperplastic tissue and most tumors, but is not expressed in normal tissue. Although the expression of Lam-5 was shown in a few glands with endocervical hyperplasia, these cells might have already acquired tumor characteristics without morphological changes i.e. precursor cells derived from endocervical glands that expressed Lam-5 might acquire tumor characteristics.

Based on our results, we suggest that Lam-5 expression might be an early event during neoplastic progression towards AC. According to our hypothesis, the upregulation of expression of Lam-5 might be related to tumor progression, e.g. the adenoma-noninvasive carcinoma-invasive carcinoma sequence seen in colon carcinogenesis. It seems clear from the literature that structural changes in Lam-5 are relatively early events during the development of invasiveness. On the other hand, the frequency of Lam-5 expression decreased, to 75% in invasive AC although it is 100% in microinvasive AC. This interpretation is difficult, but Lam-5 may be expressed at an early stage of the invasion. Furthermore the expression of Lam-5 might be attenuated in the later phases when tumor cells are more extensively invasive. Although the detailed molecular carcinogenic mechanism is unknown, it does seem to be related to Lam-5 expression and the acquirement of invasiveness. Several investigators have shown that the Lam-5 chain is expressed in epithelial cells at the invasive front of malignant tumors [9,11,12,17-23]. Other investigators also describe how Lam-5 could become a useful biomarker for the early detection of invasive tumor cells [20,29,30].

Cervical cancer is the second most common malignancy in women worldwide [31]. Of the various histologic subtypes, SCC is by far the most common form while AC is relatively rare. In recent years, epidemiologic studies have shown an increasing incidence of AC worldwide [32], especially in younger patients [1,33]. AC is a highly aggressive gynecologic malignancy. Over the last several decades, the relative proportion of AC to SCC of the uterine cervix has been increasing with several studies reporting an increase in the absolute number of AC cases [34]. In uterine cervical neoplasia, there are many etiologies that explain why the AC patient has a poorer prognosis than that of SCC [35]. One is the high tendency for invasiveness of adenocarcinoma cells. It is often hard to diagnose by colposcopy, cytodiagnosis and punch biopsy, since AC tends to grow endophytically. With regard to diagnosis, differentiation between adenocarcinoma in situ (FIGO 0), microinvasive (Ia), and invasive adenocarcinoma (Ib) is an important and difficult question to answer. The treatment strategy for patients is different for adenocarcinoma in situ, microinvasive adenocarcinoma and invasive adenocarcinoma. The patient will receive disease-specific treatments e.g. conization in adenocarcinoma in situ, simple hysterectomy in microinvasive adenocarcinoma and radical hysterectomy with additional lymphadenectomy in invasive adenocarcinoma. It is often difficult to make this decision preoperatively. We conclude in this article that the immunohistochemistry of Lam-5 is effective as a means to solve this problem. Specifically, when cells expressing Lam-5 were observed in the lesion by biopsy, we are able to surmise that those cells are invasive. Therefore a simple hysterectomy and/or more extensive surgery is indicated for these patients. In addition, even if the lesion was non-invasive in CIS morphologically, these tumor cells might have acquired invasiveness when the cells express Lam-5. In such a case, intensive follow-up care is required after treatment by conization. Thus, a sensitive diagnostic procedure is a prerequisite for appropriate therapy.

## Conclusion

It is only cases of microinvasive or invasive adenocarcinoma that have shown cytoplasmic immunoreactivity to Lam-5. These findings are similar to other reports that have compared immunoreactivity in different tumors, particularly cervical squamous neoplasia. Our results indicate that immunohistochemical expression of Lam-5 is a useful marker for detecting invasive tumor cells in AC. We can evaluate invasion in AC more precisely using Lam-5 immunohistochemistry as an adjunct to morphological examination. This method could be useful in the histopathological diagnosis of cervical cancer.

## Abbreviations

Lam-5: Laminin-5 (Laminin γ2 chain); AC: Adenocarcinoma; SCC: Squamous cell carcinoma; FIGO: International Federation of Gynecology and Obstetrics; uPAR: Urinary-type plasminogen activator receptor.

## Competing interests

No financial and non-financial competing interests to declare in relation to this manuscript.

## Authors' contributions

JI was involved in the design of the study and immunohistochemical analysis, and drafted the manuscript. YU conceived the study, was involved in the design and immunohistochemical analysis, and edited the manuscript for intellectual content. KI, ST, TI and TF were involved in the design of the study and pathological diagnosis. All authors read and approved the final manuscript.
